# Ligand sensitivity of type-1 inositol 1,4,5-trisphosphate receptor is enhanced by the D2594K mutation

**DOI:** 10.1007/s00424-023-02796-x

**Published:** 2023-03-07

**Authors:** Allison Tambeaux, Yuriana Aguilar-Sánchez, Demetrio J. Santiago, Madeleine Mascitti, Karyn M. DiNovo, Rafael Mejía-Alvarez, Michael Fill, S. R. Wayne Chen, Josefina Ramos-Franco

**Affiliations:** 1 Department of Physiology and Biophysics, Rush University Medical Center, Chicago, IL USA; 2 Present Address: Molecular Physiology & Biophysics, Baylor College of Medicine, Houston, TX USA; 3 Present Address: Centro Nacional de Investigaciones Cardiovasculares, Madrid, Spain; 4 Department of Physiology, Midwestern University, Downers Grove, IL USA; 5 Department of Physiology and Pharmacology, Libin Cardiovascular Institute, University of Calgary, Calgary, AB Canada

**Keywords:** Inositol 1,4,5-trisphosphate receptor, IP_3_, Gain of function, Ca^2+^ puffs, Single channels

## Abstract

Inositol 1,4,5-trisphosphate receptor (IP_3_R) and ryanodine receptor (RyR) are homologous cation channels that mediate release of Ca^2+^ from the endoplasmic/sarcoplasmic reticulum (ER/SR) and thereby are involved in many physiological processes. In previous studies, we determined that when the D2594 residue, located at or near the gate of the IP_3_R type 1, was replaced by lysine (D2594K), a gain of function was obtained. This mutant phenotype was characterized by increased IP_3_ sensitivity. We hypothesized the IP_3_R1-D2594 determines the ligand sensitivity of the channel by electrostatically affecting the stability of the closed and open states. To test this possibility, the relationship between the D2594 site and IP_3_R1 regulation by IP_3_, cytosolic, and luminal Ca^2+^ was determined at the cellular, subcellular, and single-channel levels using fluorescence Ca^2+^ imaging and single-channel reconstitution. We found that in cells, D2594K mutation enhances the IP_3_ ligand sensitivity. Single-channel IP_3_R1 studies revealed that the conductance of IP_3_R1-WT and -D2594K channels is similar. However, IP_3_R1-D2594K channels exhibit higher IP_3_ sensitivity, with substantially greater efficacy. In addition, like its wild type (WT) counterpart, IP_3_R1-D2594K showed a bell-shape cytosolic Ca^2+^-dependency, but D2594K had greater activity at each tested cytosolic free Ca^2+^ concentration. The IP_3_R1-D2594K also had altered luminal Ca^2+^ sensitivity. Unlike IP_3_R1-WT, D2594K channel activity did not decrease at low luminal Ca^2+^ levels. Taken together, our functional studies indicate that the substitution of a negatively charged residue by a positive one at the channels’ pore cytosolic exit affects the channel’s gating behavior thereby explaining the enhanced ligand-channel’s sensitivity.

**Supplementary Information:**

The online version contains supplementary material available at 10.1007/s00424-023-02796-x.

## Introduction

The endoplasmic/sarcoplasmic reticulum (ER/SR) have specialized Ca^2+^ release channels that mediate intracellular Ca^2+^ signals, which regulate many cellular processes as diverse as fertilization, apoptosis, muscle contraction, transcription, secretion, learning, and memory [1, 2, 29]. These Ca^2+^ release channels are the inositol 1,4,5-trisphosphate receptors (IP_3_Rs) and ryanodine receptors (RyRs), each having 3 different isoforms. Although in most cells, a mix of the 3 isoforms is present, there are some tissues where only one type predominates. For example, the type 1 IP_3_R (IP_3_R1) is the predominant intracellular Ca^2+^ release channel in cerebellum [23, 33], while the type 2 RyR (RyR2) predominates in the heart [32, 34]. The IP_3_Rs and RyRs share a high degree of functional and structural homology [28, 30, 41, 42, 45] which can be exploited to advance our understanding of these channels.

Production of cytosolic IP_3_ occurs when extracellular ligands (e.g., hormones, neurotransmitters) bind to Gq-protein–coupled receptors in the surface membrane to initiate an intracellular signaling cascade [1, 23, 36]. IP_3_R channel activity is primarily regulated by the availability of IP_3_ and Ca^2+^ [5, 27, 37, 38]. Specifically, single-IP_3_R1 channels are activated when they bind IP_3_, and the extent of IP_3_R1 activation (i.e., opening duration/frequency) is a bell-shaped function of the cytosolic-free Ca^2+^ concentration [5, 7, 24, 37, 38]. The bell-shaped cytosolic Ca^2+^ dependency of the IP_3_R1 is explained by the presence of a cytosolic Ca^2+^ activation site and a putative lower affinity cytosolic Ca^2+^ inhibition site [20, 31, 43]. An intense research topic of single IP_3_R1 Ca^2+^ control has been the mechanism by which intra-ER Ca^2+^ modulates the channel activity [4, 18, 44, 47, 48]. Specifically, the controversial mechanisms that have been investigated are (a) intra-ER (luminal) Ca^2+^ interaction with a luminal regulatory site on the channel itself [8, 44, 48], or (b) on a closely associated regulatory protein [15, 47, 49], and/or (c) luminal Ca^2+^ flowing through open IP_3_Rs (lumen-to-cytosol) acting on the IP_3_R’s cytosolic Ca^2+^ activation and/or Ca^2+^ inhibitory sites [4, 17, 49, 50]. This latter process is commonly referred to as feedthrough (FT) Ca^2+^ regulation. Overall, the IP_3_R1 ligand regulatory scenario involves allosterically interacting inputs [24–26, 53]. For example, Mak et al. (1998) proposed that cytosolic IP_3_ allosterically “tunes” cytosolic IP_3_R Ca^2+^ inhibition. Likewise, cytosolic ATP is a complementary allosteric regulator of IP_3_R gating [26, 48].

Single RyR activity is also governed simultaneously by multiple synergistically acting ligands. For example, single RyR2 function is controlled by cytosolic ATP and Ca^2+^ (via its cytosolic Ca^2+^ activation and inhibition sites) and by luminal Ca^2+^ (via Ca^2+^ binding sites in the channel and accessory proteins) [10, 14, 22, 52]. In regard to the luminal [Ca^2+^] control, Chen et al., using RyR2 site-directed mutagenesis of negatively charged residues within the channel’s permeation pathway, identified the RyR2-E4872 residue as a critical site involved in RyR2 luminal Ca^2+^ regulation [9]. Furthermore, functional and structural analyses revealed that the negatively charged RyR2-E4872 residue in one subunit forms a salt-bridge with the positively charged RyR2-R4874 residue in the neighboring subunit in the closed state. This inter-subunit network of electrostatic interactions is believed to stabilize the closed state of RyR2. Thus, a model was put forward in which Ca^2+^ binding to E4872 disrupts the electrostatic interactions with neighboring amino acids favoring the transition from closed to open state [9, 35].

**Fig. 1:**
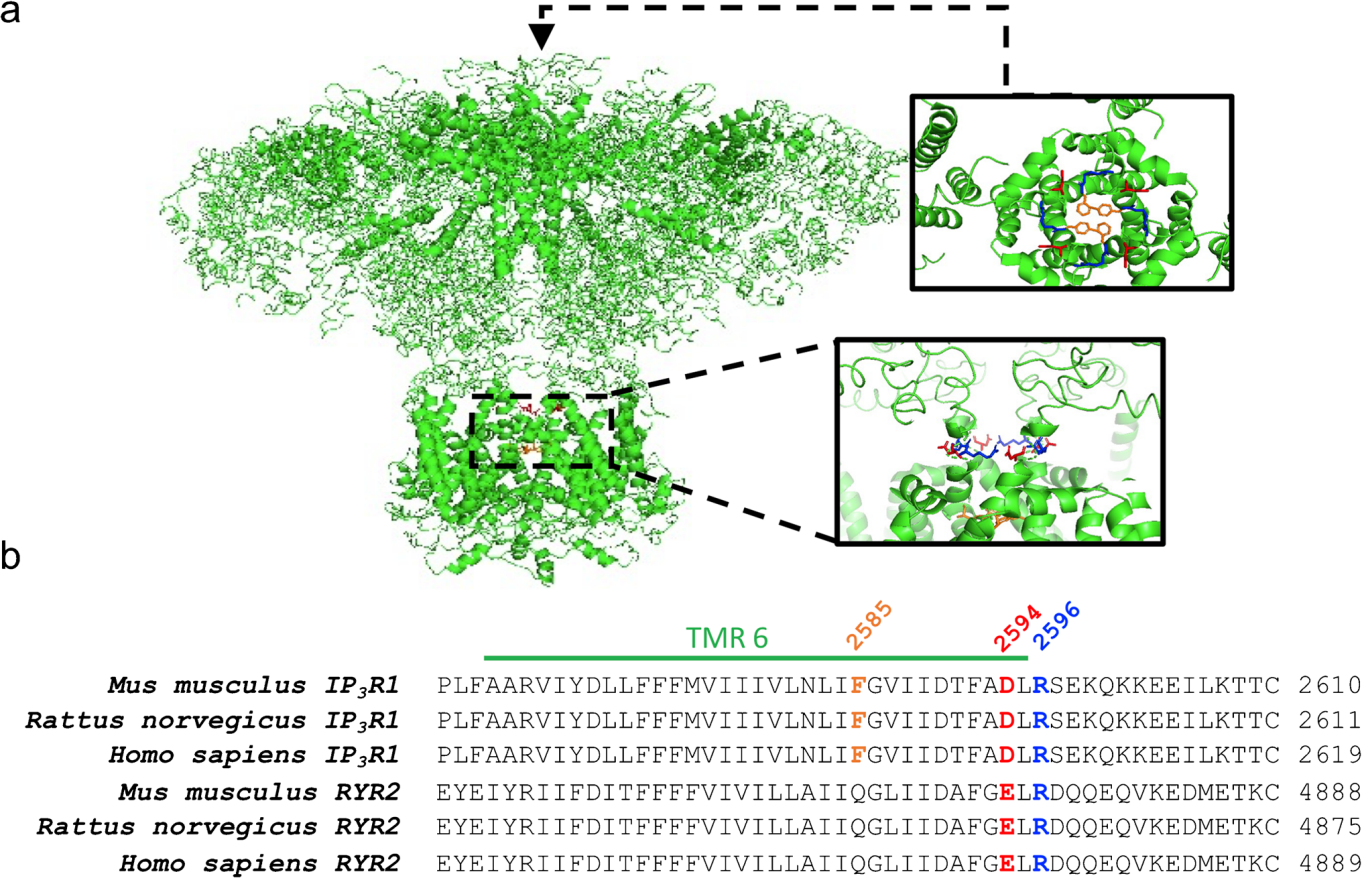
Closed IP_3_R1 channel structure. **a** Illustration of the 3D structure of IP_3_R1 (PDB ID: 3JAV; PyMOL) viewed from the cytosolic side down through the channel and along the membrane plane. The residue of interest D2594 is marked in red. Marked in blue are the conserved positively charged residues and in orange are the residues that correspond to the suggested IP_3_R1 channel’s pore gate. **b** Amino acid sequence alignment for the region containing IP_3_R1-D2594 and the corresponding site RYR2-E4872. Green line identifies the IP_3_R1 transmembrane segment 6 (aa 2564–2596) accordingly to PDB: 3JAV. Sequence alignment was done using the following IP_3_R1 sequences: *Mus musculus* (UniProt ID: P11881), *Rattus norvegicus* (UniProt ID: P29994), *Homo sapiens* (UniProt ID: Q14643). RYR2 sequences used in alignment include *Mus musculus* (UniProt ID: E9Q401), *Rattus norvegicus* (UniProt ID: B0LPN4), and *Homo sapiens* (UniProt ID: Q92736). Alignment was performed using Clustal Omega (Sievers & Higgins, 2018)

The corresponding IP_3_R1 site to RyR2-E4872 residue is the IP_3_R1-D2594 at the transmembrane region 6 (TMR6). Figure 1a shows the location of this residue in a 3D structure of the rat IP_3_R1 channel in a non-conductive closed state (4.7 Å resolution; [12]). The IP_3_R results from the assembly of four subunits arranged around the central channel axis, where two major regions can be defined, the bulky cytosolic region connected via “stalk” densities to the transmembrane regions (TMRs; [12]). The boxes show a close-up view of the ion conduction pathway, along the membrane plane (bottom) and from the cytosol (top). In orange, the F2586 residues are shown (F2585 mouse) which have been suggested to serve as the pore gate. Away from the gate, in the ion conduction pathway, the D2595 (D2594 mouse; red) and R2597 (R2596 mouse; blue) residues are marked. Similarly, to RyR2-R4874 residue, IP_3_R1-R2596 have been suggested to participate in a network of TMR interactions that could play a role in transmitting signals to the gate, in part, through an interaction between neighboring TMR6 helices [13]. Figure 1b details the amino acid sequence alignment of the region containing the IP_3_R1-D2594 and the corresponding site RYR2-E4872 in different species. Although Bhanumathy et al. did not find a substantial change in the IP_3_R1 Ca^2+^ signaling when the D2594 residue (Fig. 1, residue in red) was replaced by alanine mutagenesis [6], we have recently shown that its substitution with a positive charge (IP_3_R1-D2594K) results in a gain-of-function phenotype, while its alanine substitution (IP_3_R1-D2594A) induces a reduction of its function. To gain mechanistic insights into the functional significance of IP_3_R1-D2594K, a mouse model was created. The ITPR1‐D2594K^+/−^ mutant mice exhibited pathological ramifications that included male infertility, azoospermia, and acrosome loss [46]. These studies indicated that changing the electric nature of IP_3_R1-D2594 residue allosterically influences the IP_3_R1 channel’s activity. We speculated that changing the charge of this residue gives rise to electrostatic repulsion between side chains favoring the opening of the channel. Therefore, we hypothesized the IP_3_R1-D2594 residue determines the ligand sensitivity of the channel by electrostatically affecting the stability of the closed and open states of the channel. Consequently, the aim of the present work was to define for the first time the molecular mechanism by which the IP_3_R1-D2594K mutation induces a gain-of-function phenotype. To this end, we measured the influence of the D2594K mutation on the IP_3_ activation, cytosolic Ca^2+^ sensitivity, and luminal Ca^2+^ dependency of IP_3_R1 at the cellular, subcellular, and single-channel levels. Our results indicate that replacing this negative charge at the pore’s cytosolic exit with a positive charge (D2594K) dramatically potentiated the IP_3_R1 channel’s IP_3_, cytosolic, and luminal Ca^2+^ sensitivity. Our interpretation is that this residue influences the efficacy of cytosolic (IP_3_ and Ca^2+^) and luminal (Ca^2+^) ligands perhaps by electrostatically influencing the stability of the closed and / or open state of the channel, and thus the operation of the IP_3_R gating process.

## Materials and methods

### Generation and culture of stable inducible IP_3_R1 expressing cell lines

Briefly, as previously described [46], stable inducible WT- and D2594K-IP_3_R1 expressing HEK293 cell lines were generated using full-length ITPR1 cDNA that was sub-cloned into an inducible expression vector, pcDNA5/FRT/TO. The recombinant expression of rat IP_3_R1 was achieved by the Flp-In T-REx-293 system (Invitrogen). Cells were incubated at 37 °C under 5% CO_2_ with DMEM supplemented with 0.1 mM nonessential amino acids, 2 mM L‐glutamine, 100 units of penicillin/ml, 1% streptomycin/ml, and 5% fetal bovine serum. Cells were selected for Flp positive in 200 µg/ml hygromycin selection medium. Induction of IP_3_R1-WT or IP_3_R1-D2594K expression was initiated upon incubation with DMEM containing 1 μg/ml tetracycline.

### High throughput recording of global Ca^2+^ transients

To evaluate the changes in cytosolic Ca^2+^ concentration, stable inducible HEK293 cells were subcultured in poly-d-lysine pretreated black wall/clear bottom 96-well plates and incubated at 37 °C under 5% CO_2_. Each 96-well plate was split to have IP_3_R1-WT and -D2594K seeded cells. For the corresponding IP_3_R1 expression, after 24 h, cells were induced with tetracycline (1 µg/ml). On the day of the experiment, the media was removed and replaced with Fluo-8 NW (ATT Bioquest) solution containing 10 × pluronic F127 Plus and KRH solution (2 mM Ca^2+^, 125 mM NaCl, 5 mM KCl, 6 mM glucose, 1.2 mM MgCl_2_, 25 mM HEPES, pH 7.4). After 60-min incubation at room temperature, Fluo-8 NW solution was removed, and each well was rinsed twice with KRH 0 Ca^2+^ solution. KRH 0 Ca^2+^ solution was added back to each well, and the 96-well plate was placed into FLIPR® Tetra High Throughput Cellular Screening System (Molecular Devices). Each well on the plate was simultaneously challenged with KRH 0 Ca^2+^ solution plus different concentrations of ATP (Sigma-Aldrich) to induce Ca^2+^ release via IP_3_R1s. LED excitation wavelength of 490 nm and a 520-nm bandpass emission filter were used to acquire images. The images were captured at every 2 or 4 s. Each determination was done by duplicate at 3 different days. The resulting Fluo-8 NW fluorescent signals were measured for each well using ScreenWorks software (Molecular Devices).

### Linescan confocal microscopy imaging of Ca^2+^ puffs

Ca^2+^ puffs were recorded with linescan confocal microscopy in tetracycline-induced HEK293 cells expressing either IP_3_R1-WT or IP_3_R1-D2594K. Cells were plated on glass bottom (0.13–0.17 mm thick) plates and induced for 48 h. On the day of the experiment, cells were incubated for 30 min in normal KRH external solution containing 2 mM Ca^2+^ for ER loading, followed by 45-min incubation in the dark and at room temperature in 1 ml KRH solution containing the fluorophore Cal-520 AM (AAT Bioquest). Cal-520 AM 45 µM stock solution was prepared by dissolving 50 µg of Cal-520 AM in 20% pluronic acid in DMSO (Molecular Probes) in KRH solution. The incubation period was ended by rinsing the cells with dye-free normal external solution at room temperature for 15 min. To record Ca^2+^ puffs, Cal-520 was excited with the 488 nm line of an argon laser, and the emitted fluorescence was collected through a 515-nm long pass emission filter. Cal-520 images were recorded in bidirectional linescan mode with 256 × 512 pixels per frame at 4 Hz and *x*-time series images at 10 Hz. For consistency, all linescan confocal microscope settings were kept the same for all experiments (laser power: 5%, photomultiplier gain: 140, pin size: 0.9, pixel size: 0.63). All experiments started with perfusion with external KRH 0 Ca^2+^ solution for 1 min followed by 2-min perfusion of 20 nM ATP. Fluorescence images were analyzed with Elements v4.4 (Nikon) and ImageJ v1.52 (NIH) software. Briefly, Ca^2+^ puffs were interactively detected as areas of high fluorescence compared with the standard deviation (threshold arbitrarily set between 5 and 6.5 standard deviations) of the background fluorescence. Puff duration was measured as the dwell time at the 50% of the peak amplitude (full-duration-half-maximum; FDHM). Puffs frequency was expressed in Hz as number of Ca^2+^ puffs per cell, while their kinetic attributes were estimated by measuring time to peak (time from baseline to maximal), rise time (time from 20 to 80% maximal amplitude), and decaying time (from 80 to 20% maximal amplitude; Lock et al., 2015). All puff parameters were measured, pooled, and averaged for either IP_3_R1-WT or IP_3_R1-D2594K.

### Isolation of microsomal vesicles

Crude microsomes were obtained from control (WT), ITPR1‐D2594K^+/−^ and -D2594A^+/−^ mutant knock-in (KI) mice models developed previously [46]. Mouse cerebellar microsomes were prepared as described previously [51] with some modifications. Briefly, cerebella were homogenized in a glass-Teflon Potter homogenizer in solution A (10 mM Tris-maleate, pH 6.8, 0.5 mM DTT, and protease inhibitor cocktail; Roche cOmplete EDTA free; Sigma-Aldrich). The homogenate was centrifuged for 10 min at 4000 × gmax. The supernatant was re-centrifuged for 5 min at 15,000 × gmax, and the pellet was resuspended in solution B (10 mM Tris-Maleate, 100 mM KCl, pH 6.8, and 0.5 mM DTT). Then, the supernatant was centrifuged for 90 min at 165,000 × gmax, and the resulting pellet was resuspended in an appropriate volume of solution B. All pellets were frozen in liquid nitrogen and stored at − 80 °C.

### Single-channel recordings

Reconstitution of IP_3_R1 channels into artificial planar lipid bilayers was performed as previously described [39]. Briefly, planar lipid bilayers were formed on ~ 100-µm-diameter hole in Teflon septa, separating two compartments: cis and trans. Bilayers were formed using a 5:3:2 mixture of phosphatidylethanolamine, phosphatidylserine, and phosphatidylcholine (Avanti Polar Lipids) dissolved in decane to a final concentration of 50 mg/ml. Microsomes were added to the solution in cis that was held at ground potential. IP_3_-gated channel activity was measured at room temperature in the presence of 2 mM ATP-Tris and various concentrations of IP_3_ using Cs^+^ as current carrier. The trans side of the bilayer mimics the luminal (intra-ER) compartment, where the voltage was applied. Solutions (unless otherwise specified) contained symmetrical 250 mM CsCH_3_SO_3_, 20 mM HEPES pH 7.4, and 1 mM EGTA (70 nM or 140 nM free Ca^2+^). Channel sidedness was determined by IP_3_ sensitivity. Stock solutions of IP_3_ and Ca^2+^ (Sigma-Aldrich) were made to obtain the desired final concentrations. The cytosolic and luminal free Ca^2+^ concentrations were calculated by MaxChelator [3]. Calculations of total Ca^2+^ needed to obtain a specified free Ca^2+^ concentration were based on the presence of 1 mM EGTA for the determination of cytosolic Ca^2+^ and IP_3_ dependency. For the luminal Ca^2+^ dependency, 0.5 mM EGTA, 0.5 mM BAPTA was used in the cis solution while trans solution contained 0.5 mM EGTA, 0.5 mM BAPTA, and 0.5 mM dibromo-BAPTA. Unitary current was processed by a patch-clamp amplifier (Axopatch 200B, Axon Instruments) that was connected, via Ag/AgCl electrodes, to the cis and trans solutions through agar bridges. Data were filtered at 2 kHz and digitized at 20 kHz for computer analysis using pClamp 10 (Axon Instruments). Opening events were detected from the filtered records by the half-amplitude threshold crossing technique [11]. Events with durations < 300 µs were not included in the analysis. Channel open probability was measured from the idealized records longer than 3 min. The number of channels in each experiment was estimated from the maximal number of stacked opening events observed in the bilayer and corroborated in the total amplitude histogram of the raw data by the presence of multiple peaks [16]. IP_3_R1 channel currents depicted as positive (upward deflections of the current) reflect cation flux from the trans (luminal) to the cis (cytosolic) compartment. Likewise, the downward deflections of the current reflect the cation flux in opposite direction. Single-channel gating kinetics analysis was done by constructing dwell-time histograms for the open and closed times and fitted with the corresponding probability density functions. Mean open and closed times were calculated as the sum of rate constants exiting each gating state [11].

### Statistics

All studies were designed so that only one parameter was changed at a time. To test for differences between groups, we used Student’s *t* test (two‐tailed with Welch correction), and the two-way ANOVA with Šídák’s post hoc correction using GraphPad Prism software. Data is reported as mean ± SEM. Differences were considered statistically significant at *p* < 0.05.

## Results

### Effects of IP_3_R1-D2594K on intracellular Ca^2+^ dynamics

**Fig. 2:**
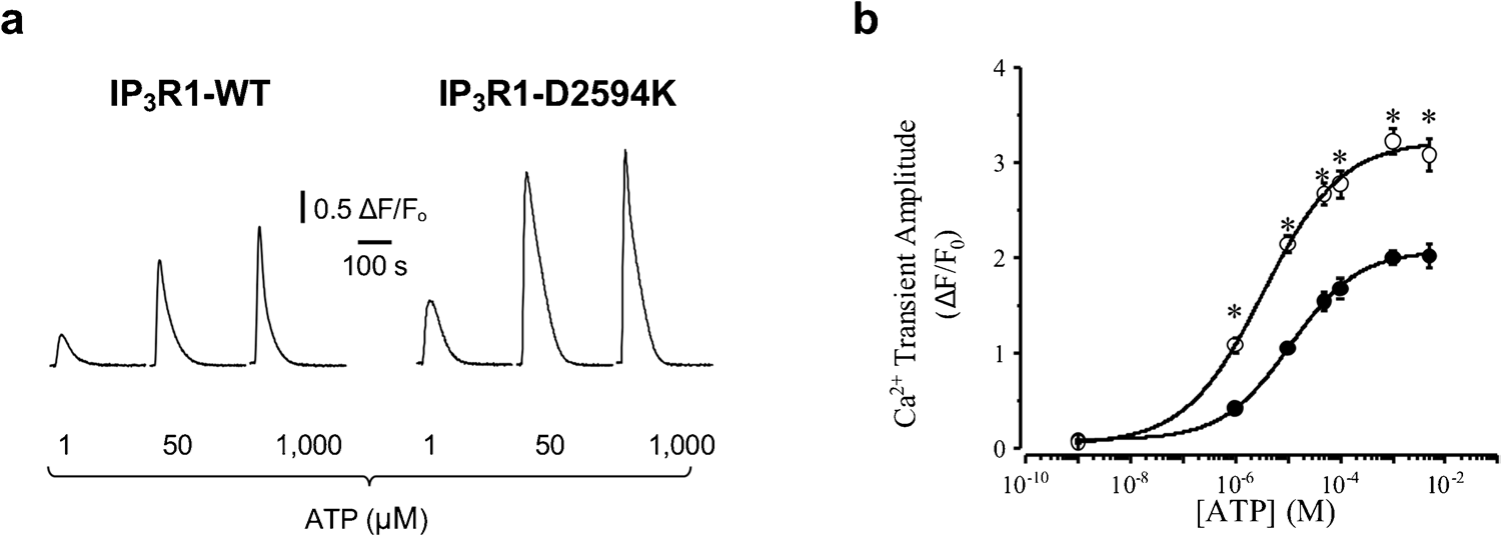
Concentration-dependent Ca^2+^ release induced by ATP. **a** Traces show typical intracellular Ca^2+^ transients in HEK 293 cells expressing IP_3_R1-WT (left) or IP_3_R1-D2594K (right) evoked by the indicated ATP concentrations. **b** Maximal Ca.^2+^ transient amplitude plotted as a function of [ATP] from cells expressing IP_3_R1-WT (filled circles) and IP_3_R1-D2594K (open circles). Data were fitted by a Hill equation with an EC_50_ of 10.9 ± 0.75 and 3.3 ± 0.7 µM for WT and mutant IP_3_R1, respectively (*n* = 6 independent plates for each [ATP]). Data points are mean ± SEM. Significance was determined using unpair *t* test *p value 0.0002 and two-way ANOVA with Šídák test *p* < 0.0001

To evaluate the functional impact of IP_3_R1 mutations on the cellular Ca^2+^ signaling, the IP_3_R1-WT and IP_3_R1-D2594K were expressed in HEK 293 cells. IP_3_R-mediated response was induced through the production of endogenous IP_3_ by stimulating Gq-protein-coupled purinergic receptors in the surface membrane with ATP. Intracellular Ca^2+^ signals were recorded with automated fluorescence plate reader in Fluo-8 NW-loaded cells. Figure 2a compares averaged fluorescence Ca^2+^ signals evoked by ATP with no added Ca^2+^, in cells expressing IP_3_R1-WT or IP_3_R1-D2594K. The evoked Ca^2+^ transients were larger in cells harboring the mutated IP_3_R channel compared to the WT. Figure 2b shows pooled measurements of intracellular Ca^2+^ transient amplitude. Maximal change in fluorescence expressed as Δ*F*/*F*_0_ is plotted as a function of the extracellular ATP stimulus (WT filled, mutant open circles). It can be observed that cells expressing IP_3_R1-D2594K produced a significantly larger signal at each [ATP] tested. The reproducibility of this response was examined by performing agonist dose–response curves by duplicate in three separate experiments. ATP stimuli greater than 1 µM evoked a significantly larger Ca^2+^ release transients when the mutant IP_3_R1 was expressed.

### Effects of IP_3_R1-D2594K on Ca^2+^ puffs

**Fig. 3:**
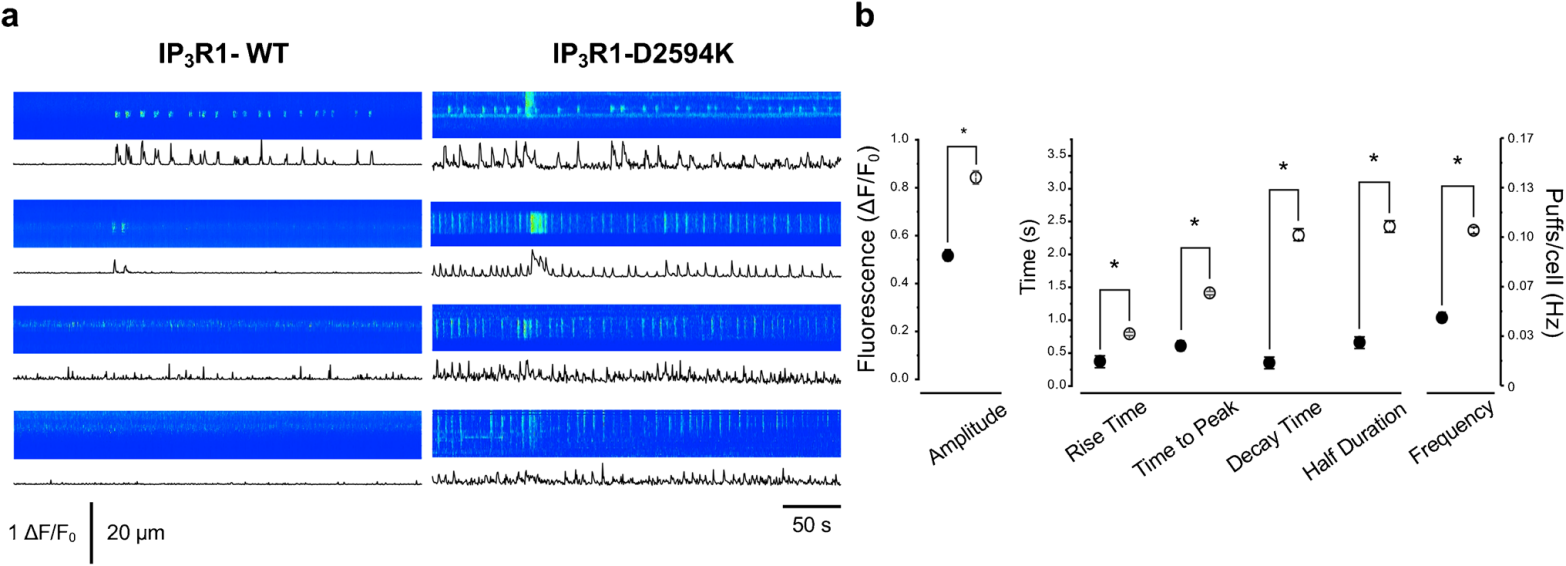
Elementary Ca^2+^ release events induced by ATP. **a** Ca^2+^ puffs recorded from HEK293 cells expressing IP_3_R1-WT or IP_3_R1-D2594K during perfusion of 0 Ca^2+^ KRH external solution containing 20 nM ATP. Fluorescence kymograph images and traces show Ca^2+^ puffs time course and frequency. **b** Pool data comparison of Ca^2+^ puffs amplitude, kinetic attributes, and frequency of occurrence evaluated from averaged traces recorded from HEK293 cells expressing either IP_3_R1-D2594K (open circles; *n* = 64 cells) or IP_3_R1-WT (filled circles; *n* = 60 cells). Data points are mean ± SEM, unpair *t* test with Welch’s correction had a *p* < 0.0001

To determine if local Ca^2+^ release events are altered by the mutated IP_3_R1, Ca^2+^ puffs were imaged in intact HEK cells expressing IP_3_R1-WT or IP_3_R1-D2594K. IP_3_R-mediated Ca^2+^ puffs are analogous to RyR-mediated Ca^2+^ sparks in striated muscle. Compared to the global Ca^2+^ transients, the attributes of Ca^2+^ puffs reflect closely the molecular IP_3_R functional properties, such as unitary conductance and single-channel gating (opening/closing) kinetics. Ca^2+^ puffs were promoted by addition of 20 nM of extracellular ATP. Our dose–response titration experiments ranging from 10 to 100 nM indicated that 20 nM ATP consistently promoted Ca^2+^ puffs and rarely induced global intracellular Ca^2+^ transients. Note these local Ca^2+^ signals are not the result of extracellular Ca^2+^ entry because the extracellular solution contained 0 mM Ca^2+^. Ca^2+^ puffs were measured during 2-min periods that were preceded and followed by control periods (no extracellular ATP present). Figure 3a shows example line scan images of Ca^2+^ puffs recorded in cells expressing IP_3_R1-WT or IP_3_R1-D2594K channels. Confocal images obtained under these conditions indicate that HEK cells expressing IP_3_R1-D2594K channels exhibit substantially higher Ca^2+^ puff activity elicited by ATP than those expressing IP_3_R1-WT. Subcellular areas with high Ca^2+^ puff activity were identified and selected as regions of interests (ROIs). In these selected regions, *x*-time images were generated with 10-µm kymograph lines to define Ca^2+^ puffs’ properties, which are better visualized by their fluorescence intensity profile plotted under each *x*-time image. From this type of fluorescent traces, Ca^2+^ puff kinetic attributes (i.e., rise time, time to peak, decay time, and half duration) were measured. Frequency of occurrence was directly quantified from selected ROIs in the *x–y* linescan images. Figure 3b reveals that IP_3_R1-D2594K-expressing cells showed more than 2.5-fold higher Ca^2+^ puffs frequency (in Hz, 0.1 ± 0.002 vs 0.04 ± 0.004 puffs/cell), while the mutant puffs’ kinetics exhibited significantly longer decay (2.28 ± 0.09 vs 0.35 ± 0.01 s), longer half duration (2.39 ± 0.09 vs 0.68 ± 0.02 s), longer time to peak (1.43 ± 0.04 vs 0.57 ± 0.02 s), and longer rise time (0.76 ± 0.03 vs 0.35 ± 0.01 s) than their counterpart from WT. To evaluate the contribution of HEK293 cells endogenous IP_3_Rs to Ca^2+^ release events, IP_3_R1-D2594K and IP_3_R1-WT cells were not induced with tetracycline, and linescan confocal images were recorded during application of external ATP. Under these conditions, infrequent or no intracellular Ca^2+^ signals were recorded in either cell type, indicating that the activity of endogenous IP_3_R was not detectable. Overall, these cellular results (Figs. 2 and 3) indicate the IP_3_R1-D2594K exhibits a gain of function phenotype.

### Effects of IP_3_R1-D2594K on unitary currents

**Fig. 4:**
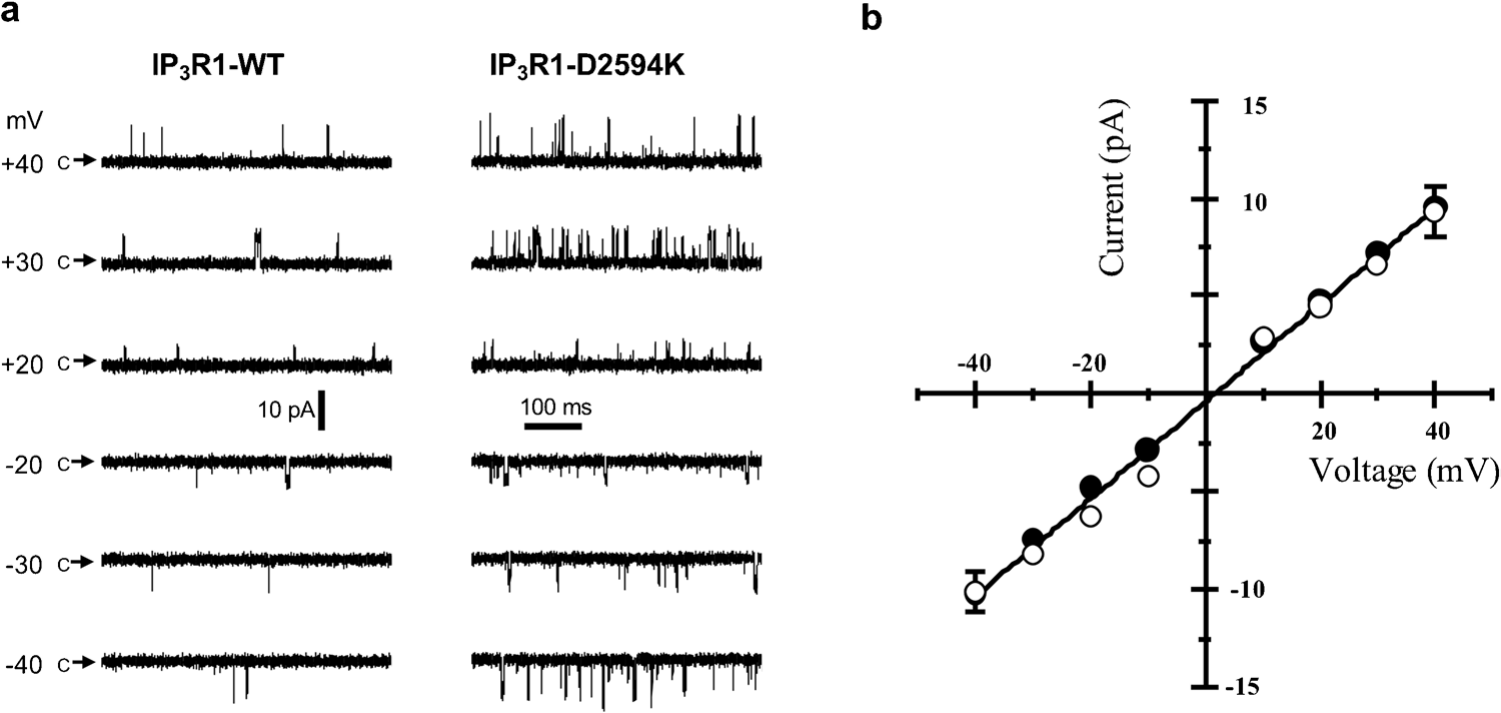
Ion conductance of single IP_3_R1-WT and IP_3_R1-D2594K channels. Symmetrical solutions contained (in mM): 250 CsCH_3_SO_3_, 1 EGTA, 0.00014 free Ca^2+^, 10 HEPES, and pH 7.4. Solution in the cytosolic side of the channel also contained (in mM) 0.010 IP_3_ and 2 ATP. **a** Sample single WT and mutant (D2594K) IP_3_R1 recordings obtained at the indicated membrane potentials. Arrows labelled “c” mark the closed non-conducting state. **b** Pooled data (mean ± SEM; *n* = 3–8) of unitary current from WT (filled circles) and D2594K (open circles) IP_3_R1 plotted as a function of membrane potential and fitted by linear regression with a conductance of 248 ± 5 pS

Since these studies were conducted in a recombinant heterologous system, it was possible that the augmented responses observed with the IP_3_R1-D2594K mutant could have resulted from an increased receptor expression. To address this possibility, IP_3_R1-WT and -D2594K single-channel activity was evaluated from our WT and KI mice. The corresponding IP_3_R1 microsomal fractions from cerebellum were reconstituted in planar lipid bilayers, and single-channel activity was studied under control conditions. As shown in Fig. 4a, the current amplitude as a function of voltage for the mutant channel was very similar to that observed in the WT. Using Cs^+^ as charge carrier, the unitary conductance of 248 ± 5 pS was not significantly different between the IP_3_R1-WT and the IP_3_R1-D2594K channels (Fig. 4b). Like IP_3_R1-WT, the IP_3_R1-D2594K Cs^+^ conductance was reduced when Ca^2+^ flux was favored by increased luminal [Ca^2+^] (see Fig. 8a and Online Resource 1), being consistent with the anomalous mole fraction effect previously described for IP_3_R1 [4].

### Effects of IP_3_R1-D2594K on IP_3_ sensitivity

**Fig. 5:**
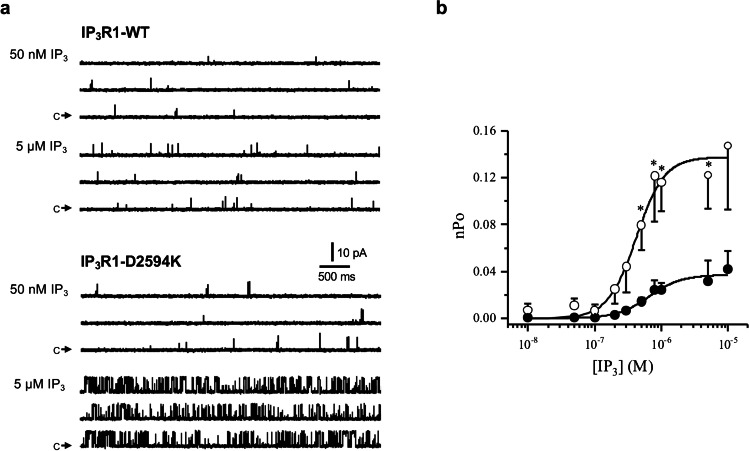
Cytosolic IP_3_ sensitivity of single IP_3_R1-WT and IP_3_R1-D2594K channels. **a** Cytosolic and luminal solutions contained symmetrical 250 mM CsCH_3_SO_3_, 1 mM EGTA, 140 nM free Ca^2+^, pH 7.4, with 2 mM ATP and the indicated IP_3_ concentration in the cytosolic side. Sample single IP_3_R1-WT and IP_3_R1-D2594K recordings made at + 30 mV are shown with 50 nM or 5 µM cytosolic IP_3_ present. Arrows labelled “c” mark the closed non-conducting state. Upward deflections represent channel openings. **b** Open probability expressed as nPo of IP_3_R1-WT and IP_3_R1-D2594K channels plotted as a function of log [IP_3_]. Pooled data (mean ± SEM; *n* = 3–9) were fitted by a Hill equation with an EC_50_ of 660 amf 406 nM and a Hill coefficient of 1.8 and 2 for IP_3_R1-WT (filled circles) and IP_3_R1-D2594K (open circles), respectively. Statistical significance between WT and mutant data points was evaluated with an unpaired *t* test with *p* < 0.05 and two-way ANOVA with Šídák test *p* < 0.0001

To gain insight about the mechanism for the enhanced response to ATP by cells expressing IP_3_R1-D2594K, its ligand sensitivity was determined at the single-channel level. Figure 5 compares the cytosolic IP_3_ sensitivity of the WT and mutant IP_3_R1 channels. Representative single IP_3_R1 recordings with 50 nM and 5 µM IP_3_ present are shown (Fig. 5a). The frequency of WT and mutant channel openings is higher at 5 µM IP_3_ (compared to 50 nM). However, the mutant IP_3_R1 is far more active (compared to WT) at 5 µM IP_3_. Figure 5b plots pooled open probability normalized by the number of channels present in the bilayer (expressed as nPo) of WT and mutant channels as a function of cytosolic [IP_3_]. These data were well fitted by a Hill equation with significantly different EC_50_ values (660 nM for WT and 406 nM for mutant). In addition, IP_3_ activated the mutant IP_3_R to a significantly higher Po level. The maximal Po reached at 10 µM IP_3_ was 3.6-fold higher for the IP_3_R1-D2594K compared to the WT channel. Thus, the IP_3_R1-D2594K mutation increased the sensitivity and efficacy of IP_3_ activation.

### Effects of IP_3_R1-D2594K on cytosolic Ca^2+^ sensitivity

**Fig. 6:**
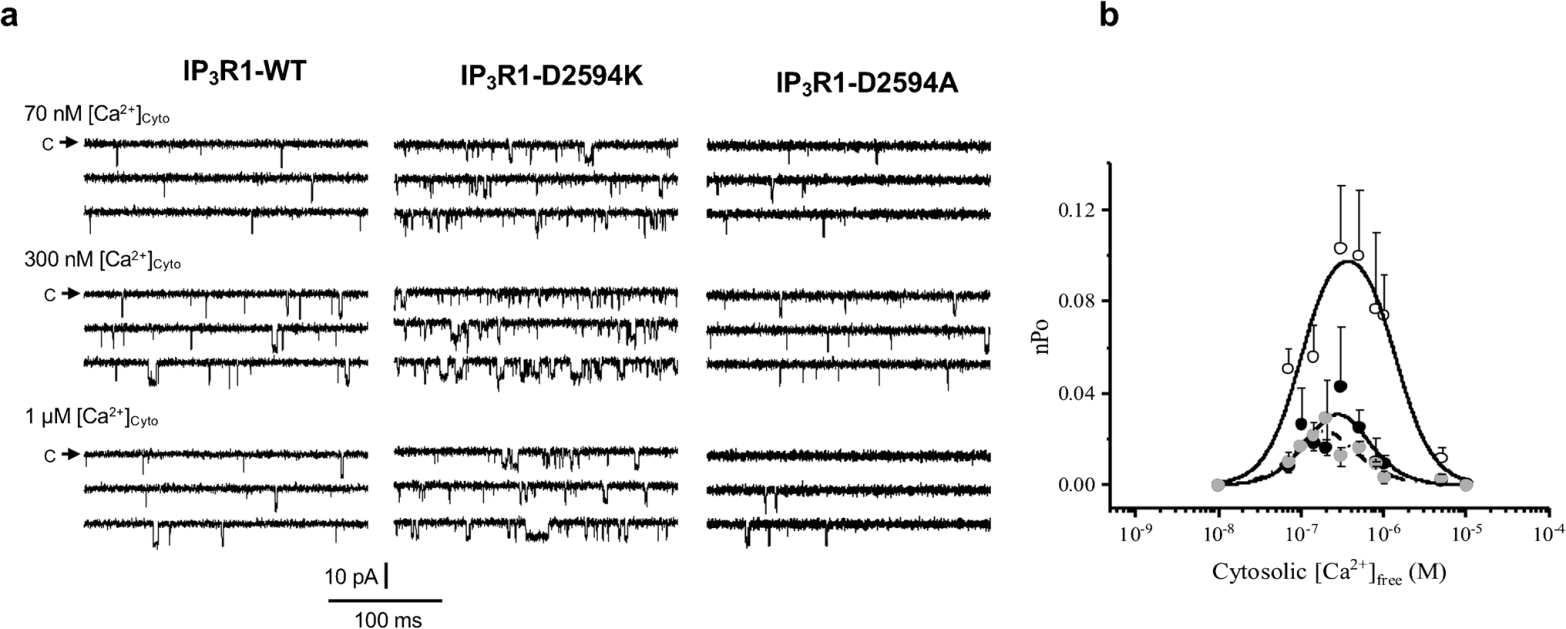
Cytosolic Ca^2+^ dependency of IP_3_R1-WT, IP_3_R1-D2594K, and IP_3_R1-D2594A. The cytosolic and luminal solutions respectively contained (in mM): 500/50 CsCH_3_SO_3_, 1/1 EGTA, various/0.00007 [Ca^2+^]_free_, 2/0 ATP, 0.01/0 IP_3_ at pH 7.4. **a** Single-channel activity from IP_3_R1-WT (left), IP_3_R1-D2594K (center), and IP_3_R1-D2594A (right), recorded at 0 mV at the indicated cytosolic [Ca^2+^]. Downward deflections indicate channel openings. **b** Open probability as a function of Log10 cytosolic [Ca.^2+^] for IP_3_R1- WT (filled circles), IP_3_R1-D2594A (gray circles), and IP_3_R1-D2594K (open circles). Pooled data shown as mean ± SEM (*n* = 9–12) were fitted with a biphasic Hill equation. Significance was determined using ANOVA with Šídák test with *p* values 0.006 (WT vs. D2594K) and 0.962 (WT vs. D2594A)

In a previous study, we found that when the IP_3_R1-D2594 residue was replaced by alanine, the concentration-dependent response to IP_3_ was reduced, while replacing it by a lysine or arginine, the response was substantially increased [46]. One possible mechanism to explain these effects is that the D2594 residue determines the channel sensitivity to cytosolic Ca^2+^. To evaluate this possibility, the experiments shown in Fig. 6 compare the cytosolic Ca^2+^ sensitivity of single IP_3_R1-WT with that of D2594 mutated IP_3_R1 channels. Representative single IP_3_R1 channel activity obtained in 70 nM, 300 nM, and 1 µM cytosolic Ca^2+^ is shown (Fig. 6a). At 70 nM cytosolic Ca^2+^, openings of all three IP_3_R1 channels are brief and relatively rare, but for IP_3_R1-WT and IP_3_R1-D2594K, they become longer and more frequent when the cytosolic Ca^2+^ is increased to 300 nM. Further increasing the cytosolic Ca^2+^ to 1 µM reduced the activity of these three types of IP_3_R1 channels. Still, the activity of the IP_3_R1-D2594K channel at all tested cytosolic Ca^2+^ levels was considerably greater than the WT channel. Although the IP_3_R1-D2594A activity also exhibited a bell-shaped dependency, the opening events remained brief and scarce at the three cytosolic Ca^2+^ concentrations. Figure 6b plots nPo of IP_3_R1-WT, IP_3_R1-D2594K, and IP_3_R1-D2594A channels as a function of cytosolic Ca^2+^. These experiments were conducted in the presence of 10 μM cytosolic IP_3_ and 70 nM luminal Ca^2+^. The cytosolic Ca^2+^ dependency of all three channels exhibited a bell-shaped relationship, but the IP_3_R1-D2594K activity was considerably larger at all tested [Ca^2+^]. These data were well fitted by a biphasic Hill equation with the following EC_50_ and IC_50_ values: 78 and 562 nM for IP_3_R1-WT, 101 and 1380 nM for IP_3_R1-D2594K, and 89 and 399 nM for IP_3_R1-D2594A, respectively. Across the range of all Ca^2+^ levels tested, cytosolic Ca^2+^ activated the IP_3_R-D2594K to a significantly larger Po level. The maximal Po reached near 300 nM Ca^2+^ was roughly threefold higher for the IP_3_R1-D2594K than for the other two channel types. This shows that the D2594K mutation also increases the cytosolic Ca^2+^ activation efficacy without substantially altering the channel’s cytosolic Ca^2+^ sensitivity given the similarity of EC_50_ values.

### Effects of IP_3_R1-D2594K on luminal Ca^2+^ sensitivity

**Fig. 7:**
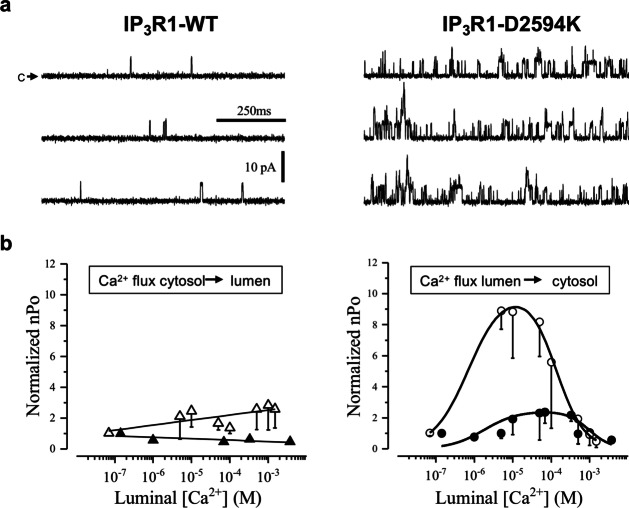
Luminal Ca^2+^ sensitivity of single IP_3_R1-WT and IP_3_R1-D2594K channels. The cytosolic and luminal solutions respectively contained (in mM) 250/250 CsCH_3_SO_3_, 0.5/0.5 EGTA, 0.5/0 BAPTA, 0.00007/various [Ca^2+^]_free_, 0.01/0 IP_3_, 2/0 ATP and pH 7.4. **a** Sample single IP_3_R1-WT and IP_3_R1-D2594K recordings made at + 30 mV with 10 µM luminal free Ca^2+^ present. Arrow labelled “c” mark the closed non-conducting state. Upward deflections represent channel openings. **b** Left, IP_3_R1-WT (filled triangles) and -D2594K (open triangles) nPo plotted as a function of luminal [Ca^2+^] at negative applied voltages. Data are mean ± SEM (*n* = 3–9) and were fitted by linear regression. **b** Right, nPo of IP_3_R1-WT (filled circles) and -D2594K (open circles) plotted as a function of luminal [Ca.^2+^] at positive applied voltages. Data are mean ± SEM (*n* = 4–9) fit by a biphasic Hill equation. Statistical significance at positive potentials by unpaired *t* test with Welch’s correction had a *p* value 0.0015

The mechanism of luminal Ca^2+^ regulation has been the focus of intense research [4, 8, 18, 24, 44, 48–50]; therefore, the effect of IP_3_R1-D2594K on this property was investigated. Figure 7a shows sample single IP_3_R1-WT and IP_3_R1-D2594K channel recordings with 10 µM luminal Ca^2+^, 10 µM cytosolic IP_3_, and 70 nM free cytosolic Ca^2+^. The mutant IP_3_R1 is significantly more active than the WT channel. A possible mechanism of luminal Ca^2+^ regulation would involve luminal Ca^2+^ ions feeding through (FT) an open IP_3_R1, as they move from lumen of the ER to cytosol, and binding to the channel’s own cytosolic Ca^2+^ activation and inhibition sites. Logically, the degree of Ca^2+^ regulation by FT will depend on the amplitude of Ca^2+^ flux through the open channel, which varies with both the trans-membrane Ca^2+^ gradient and the ER membrane potential. Figure 7b left compares the activity of IP_3_R1-WT (filled triangles) with IP_3_R1-D2594K (open triangles) normalized to the initial nPo, as a function of luminal [Ca^2+^] at − 40 mV of membrane potential, which favored cytosol-to-lumen Ca^2+^ flux. Under these conditions, the luminal Ca^2+^ dependency was essentially eliminated, as indicated by the straight lines fitted to the data points. In contrast, Fig. 7b right shows that at + 40 mV, which favored the lumen-to-cytosol Ca^2+^ flux, the luminal Ca^2+^ dependency of both types of channels becomes bell-shaped, resembling the cytosolic Ca^2+^ dependency. This bell-shaped behavior indicates the predominance of activation over inhibition at low luminal [Ca^2+^], and the predominant inhibitory effect as luminal [Ca^2+^] is increased. However, the activity of IP_3_R1-D2594K (open circles) as a function of luminal [Ca^2+^] was more than threefold greater than that of the WT channel (filled circles) and peaked at about one order of magnitude lower luminal [Ca^2+^] (10 vs 100 μM, respectively). These dramatic effects observed across most of luminal Ca^2+^ levels tested were unlikely due to the mutant conducting a larger flux because as shown in Fig. 4, the mutation does not alter IP_3_R1 unitary conductance.

## Discussion

IP_3_R and RyR share many structural and functional similarities. In RyR2, the negatively charged residue E4872 electrostatically interacts with the positively charged residue R4874 in the neighboring subunit to stabilize the closed state. When open, E4872 moves away from R4874, but closer to the negatively charged residue E4878. These residues (E4872 and E4878) are thought to be involved in electrostatic interaction with luminal Ca^2+^ [19, 35]. The corresponding E4872 residue in IP_3_R1 is D2594, but its role in IP_3_R function is unknown. We have previously found that when the D2594 residue’s charge was neutralized, the IP_3_ sensitivity of IP_3_R1 was reduced, but the IP_3_ sensitivity increased if it was replaced by a positive residue like lysine or arginine [46]. In this work, we found that in intact cells, this substitution increased the efficacy of activation by IP_3_ (shifted the EC_50_), while at the subcellular level, it increased the frequency, amplitude, and duration of Ca^2+^ puffs. These actions could be explained by differences in cell densities, expression levels, and/or altered receptor regulation. To address these possibilities, we defined how the D2594K alters IP_3_R1 function at the single-channel level. We showed (Fig. 5b) the mutation increased Po and shifted the IP_3_ EC_50_, indicative of an increase in the channel’s IP_3_ sensitivity. However, this could also be explained in part by changes in either IP_3_R1 cytosolic and/or luminal [Ca^2+^] regulation. We found that IP_3_R1-D2594K, like its WT counterpart, exhibited a bell-shaped cytosolic-free [Ca^2+^] dependency, but the mutant exhibited higher Po even when the lumen-to-cytosol Ca^2+^ flux was minimized by reducing the Ca^2+^ driving force with 70 nM luminal [Ca^2+^]. As seen in Fig. 6, the channel activity at both 70 and 300 nM cytosolic [Ca^2+^] was considerably higher in the mutant channel than in the WT with low luminal [Ca^2+^]. Likewise, WT and D2594K channels were both sensitive to luminal [Ca^2+^], but the mutant’s Po was higher as well. Although potential effects of D2594K on ionic permeability/selectivity were not ruled out, our Ca^2+^/Cs^+^ selectivity experiments suggest this possibility is unlikely (see Fig. 4 and Online resource 1). Taken together, it appears that this mutation near the channel’s gate simultaneously alters the IP_3_ and Ca^2+^ (cytosolic and luminal) sensitivities of the channel to produce its clear gain-of -function phenotype. This is consistent with the notion that the D2594 residue stabilizes the IP_3_R1 closed state, as it has been proposed by Ogawa’s group for the corresponding residue in RyR2, where specific interactions within the pore stabilize and prevent hyperactivity [21]. Furthermore, this possibility has already been suggested for the IP_3_R as point mutations in the channel permeation pathway alter intra/inter-domain interactions and have secondary regulatory actions [7, 40].

Specifically, we speculate that IP_3_R1 D2594 residue electrostatically interacts with positively charged R2596 in the neighboring subunit. This forms a network of salt bridges that stabilizes the IP_3_R1 closed state. This makes the R2594 residue key for IP_3_R1 stabilization and prevention of potentially deleterious hyperactivity.

**Fig. 8:**
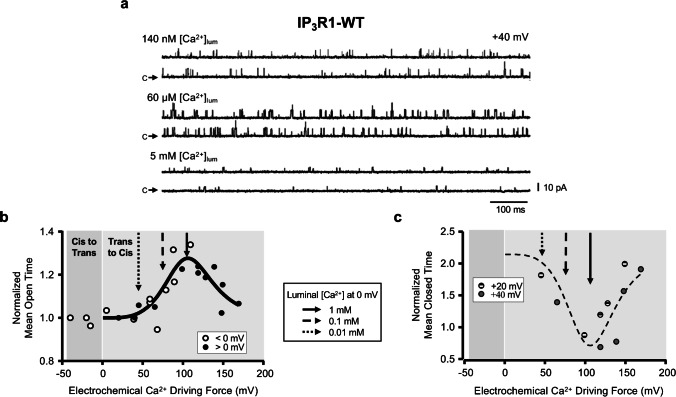
Luminal Ca^2+^ dependency of single IP_3_R1-WT channels gating kinetics. The cytosolic and luminal solutions contained symmetrical 250 mM CsCH_3_SO_3_, 1 mM EGTA, 140 nM [Ca^2+^]_free_, pH 7.4. **a** Unitary Cs^+^ currents at + 40 mV with 2 mM ATP, 2 µM IP_3_ at the indicated 140 nM (upper), 60 µM (middle), or 5 mM (lower) free luminal Ca^2+^. Label “c” marks the closed non-conducting state. Upward deflections represent channel openings. **b** Normalized mean open time of IP_3_R1-WT channel plotted as a function of the electrochemical Ca^2+^ driving force (E_m_ − E_Ca_) in mV, where *E*_*m*_ is the transmembrane potential applied to the bilayer (i.e., − 40, − 20, + 20, or + 40 mV) with the cis side held at ground and *E*_*Ca*_ is the Nernst potential of Ca^2+^ in mV, calculated at 22 ºC by the formula *E*_*Ca*_ = (*RT*/*z*Ca^2+^*F*) ln([Ca^2+^]Cis/[Ca^2+^]Trans), where *R* is the universal gas constant, 8314 mJ/(K mol); *F* is Faraday’s constant, 96,485 C/mol; *T* is temperature, 295.15 K; and zCa^2+^ is the valence of Ca^2+^ ions, + 2. With these formulas, positive electrochemical driving forces indicate trans-to-cis Ca^2+^ fluxes. Data points were measured at − 40, − 20 mV (open circles), + 20, and + 40 mV (black circles). Data fitted with a double Hill equation (MOT = 1 + Range∙(1 + (Va_50_/EDF)^Ha) − 1 (1 + (EDF/Vi_50_)^Hi) − 1) with Va_50_ = 111.5 mV, Ha = 7.8, Vi_50_ = 170.7 mV, Hi = 3.7. Arrows represent the electrochemical driving force for Ca^2+^ at *E*_*m*_ = 0 mV (the physiological transmembrane potential at the ER membrane) at full (1 mM), depleted (100 µM) or an extremely depleted (10 µM) ER, with 140 nM Ca^2+^ at the cytosol. **c** Normalized closed time of IP_3_R1-WT channel plotted as a function of the electrochemical Ca^2+^ driving force. Data points were measured at + 20 (dashed circles) and + 40 mV (gray circles). For comparison, the curve fitted to MOT (flipped and scaled) is included as a dashed line

As mentioned, the IP_3_R1-D2594 residue is homologous to RyR2-E4872 which (in RyR2) influences luminal Ca^2+^ regulation [9]. Replacing RyR2-E4872 for alanine abolished luminal Ca^2+^ dependency, but it was restored by introducing a glutamate residue nearby (G4871E). Thus, we sought to better detail the potential influence of IP_3_R1-D2594 on luminal Ca^2+^ regulation. Figure 8 shows the actions of luminal [Ca^2+^] and membrane potential on the duration of IP_3_R1-WT opening and closing events. Single IP_3_R1-WT channel activity obtained at three different luminal [Ca^2+^] at + 40 mV (Fig. 8a) illustrates the clear luminal [Ca^2+^] dependency of IP_3_R1 Po. Note the unitary Cs^+^ current is small at 5 mM luminal (as Ca^2+^ competes with Cs^+^ for occupancy of the pore). The duration of IP_3_R1 opening and closing events varied with luminal [Ca^2+^]. We analyzed mean open time (MOT) and mean closed time (MCT) separately because lumen-to-cytosol Ca^2+^ flux carried by an open channel may act on cytosolic Ca^2+^ regulatory sites and extend mean open time (MOT; [50]). Intuitively, lumen-to-cytosol Ca^2+^ flux should not influence closed channel function. In Fig. 8b, MOT was normalized (to its initial value) and plotted as a function of the electrochemical Ca^2+^ driving force (EDF, measured as the difference between the membrane potential and the Ca^2+^ Nernst potential; *E*_*m*_ − *E*_*Ca*_). Interestingly, single IP_3_R1 MOT displayed a bell-shaped dependency on EDF. As indicated by the vertical arrows, MOT decreased as the ER [Ca^2+^] is reduced. Arrows represent the expected EDFs at room temperature, for an ER that is full of Ca^2+^ (solid arrow), a depleted ER (dash arrow), and an extremely depleted ER (square dot arrow). Note that the activation phase lies within the physiological range of electrochemical driving forces for Ca^2+^. The MOT behavior is reminiscent of the IP_3_R1’s bell-shaped cytosolic Ca^2+^ sensitivity (see Fig. 6) and strongly suggests luminal Ca^2+^ feeds through an open IP_3_R1 and acts on its cytosolic Ca^2+^ activation and inactivation sites. Interestingly, the mean closed time (MCT) also exhibited a profound dependency on EDF as illustrated in Fig. 8c. In fact, the experimental data points were well described by the bell-shaped curve fitted to the MOT (double-Hill equation scaled and inverted; Fig. 8c; dashed curve). Although our results cannot definitively rule out the presence of a luminal Ca^2+^ binding site (in the channel itself and/or in an accessory protein), our analysis indicates that the IP_3_R1 dependence on luminal [Ca^2+^] is strongly influenced by the Ca^2+^ ions passing through the IP_3_R1. Unfortunately, the same type of kinetic analysis could not be performed on IP_3_R-D2594K channels because multiple (not single) mutant channels were typically present. However, based on our cytosolic and luminal [Ca^2+^] studies, we believe mutant channel dwell times would likely exhibit similar dependencies on EDF.

In summary, a ligand gated channel’s gate interacts (likely allosterically) with ligand binding sites. Our data show the intra-pore near-the-gate IP_3_R1-D2594 residue critically contributes to ligand interactions (likely allosterically) of multiple IP_3_R1 binding sites. Thus, the IP_3_R1-D2594 residue is located in an ideal position to influence gating kinetics by establishing specific interactions within the pore to stabilize and prevent hyperactivity.

## Supplementary Information

Below is the link to the electronic supplementary material.

Supplementary file1 (PDF 578 KB)

## Data Availability

The dataset generated during the present study are available from the corresponding author on reasonable request.
