# Supplementary material for: Ligand sensitivity of type-1 inositol 1,4,5-trisphosphate receptor is enhanced by the D2594K mutation
Source: Pflugers Arch. 2023 Mar 7;475(5):569–81. doi: 10.1007/s00424-023-02796-x (PMC10105685; doi:10.1007/s00424-023-02796-x)
Supplement: Supplementary file 1 — Supplementary file1 (PDF 578 KB) [file 424_2023_2796_MOESM1_ESM.pdf]

Ligand Sensitivity of Type-1 Inositol 1,4,5-Trisphosphate Receptor is Enhanced by the D2594K Mutation

Tambeaux, Allison [1], Yuriana Aguilar-Sánchez [1*], Demetrio J. Santiago [1**], Madeleine Mascitti [2], Karyn M. DiNovo [2], Rafael Mejía-Alvarez [2], Michael Fill [1], S.R. Wayne Chen[1, 3], and Josefina Ramos-Franco [1].

[1] Department of Physiology and Biophysics, Rush University Medical Center, Chicago IL, United States of America

[2] Department of Physiology, Midwestern University, Downers Grove, IL, United States of America

[3] Departments of Physiology and Pharmacology, Libin Cardiovascular Institute, University of Calgary, Calgary, Alberta, Canada

* Current address: Molecular Physiology & Biophysics, Baylor College of Medicine, Houston, TX, United States of America

** Current address: Centro Nacional de Investigaciones Cardiovasculares, Madrid, Spain

Correspondence

Josefina Ramos-Franco

jrfranco@rush.edu

Online Resources

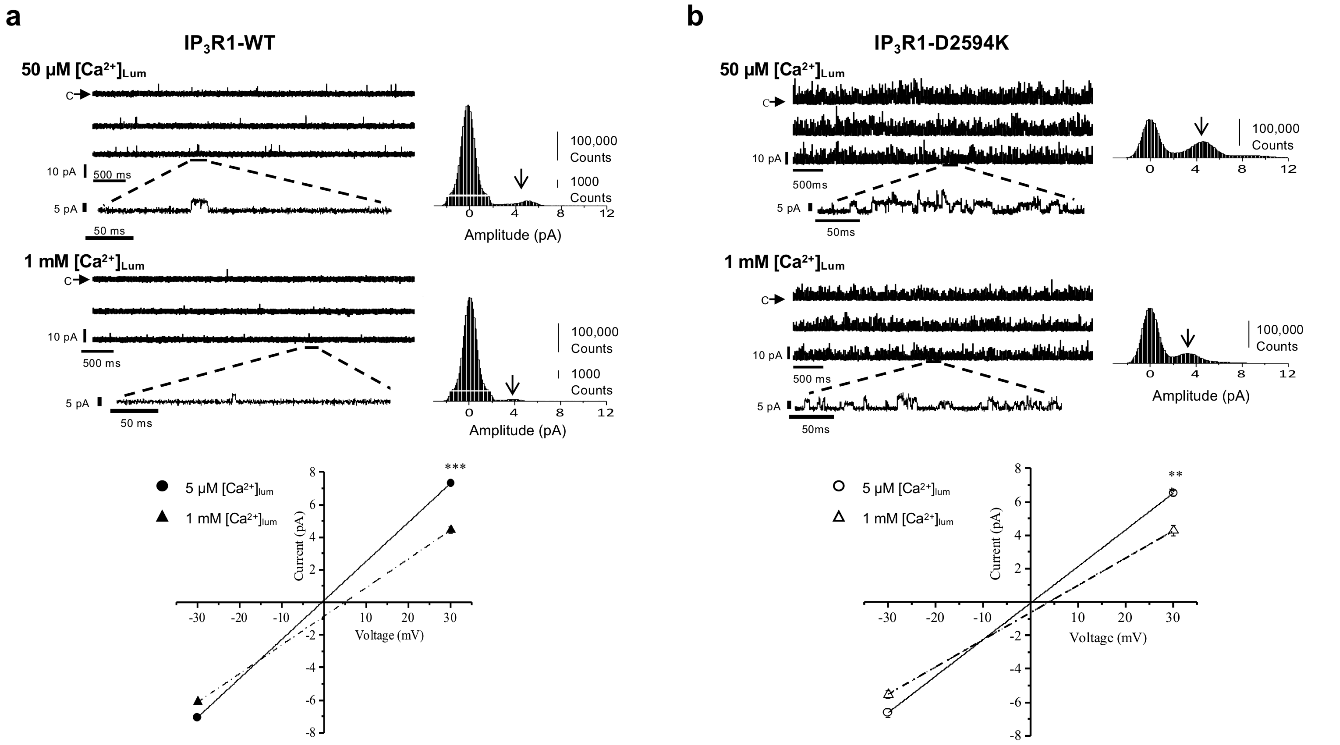

Online Resource 1 Anomalous mole fraction effect. Unitary current at the indicated luminal Ca^{2+} in the presence of symmetrical 250/250 mM $CsCH_3SO_3$, 1 mM EGTA, 10 mM HEPES pH 7.4, with 2 mM ATP,10 µM IP_3 and 70 nM free Ca^{2+} in Cis. Label "c" marks the closed non-conducting state. (a) IP_3R1-WT single-channel activity recorded at +30 mV, with Cs^+ as primary charge carrier at the indicated luminal [Ca^{2+}]. Upward deflections indicate channel openings. Three minutes total amplitude histograms correspond to the adjacent single-channel traces. Bottom, current-voltage relationships for IP_3R1-WT had a conductance of 240 pS with 5 µM Ca^{2+} (solid circles) and 176 pS with 1mM added Ca^{2+} (solid triangles). Data points were fitted by linear regression. (b) Single-channel activity from IP_3R1-D2594K recorded at +30 mV, with Cs^+ as the primary charge carrier at the indicated luminal [Ca^{2+}]. Below, respective current-voltage relationship had a conductance of 220 pS with 5 µM Ca^{2+} (open circles) and 163 pS with 1mM added Ca^{2+} (open triangles). Statistical significance between low and high added [Ca^{2+}] at positive potentials had a $p < 0.05$ (***,**).
